# Motor equivalence in motor awareness

**DOI:** 10.1016/j.isci.2026.115835

**Published:** 2026-04-21

**Authors:** Jemina Fasola, Sophie Betka, Nathan Faivre, Olaf Blanke, Oliver Alan Kannape

**Affiliations:** 1 Laboratory of Cognitive Neuroscience, Faculty of Life Sciences, Neuro-X Institute, Swiss Federal Institute of Technology (EPFL), 1012 Geneva, Switzerland; 2 University Grenoble Alpes, University Savoie Mont Blanc, CNRS, LPNC, 38000 Grenoble, France; 3 Department of Clinical Neurosciences, Faculty of Medicine, University of Geneva, Geneva, Switzerland; 4 Virtual Medicine Center, HUG-NeuroCentre, Department of Clinical Neurosciences, University Hospitals Geneva, Geneva, Switzerland

**Keywords:** psychology

## Abstract

Motor awareness (MA) describes the conscious access we have to the details of our movements. Although sensorimotor control relies on effector-specific transformations, many studies report similar MA thresholds across effectors, suggesting a shared, abstract representation. We tested this by having participants perform the same goal-directed reaching task once with a hand-held joystick and once by leaning with the whole body (center-of-mass displacement), using veridical or randomly deviated visual feedback. We measured corrective motor responses and fitted psychometric functions to derive MA thresholds. MA did not differ between effectors in non-deviated control trials or in “converging” trials where compensation aided the reach and thresholds correlated strongly between effectors. By contrast, in “diverging” trials with increased kinematic demand, MA relied more on effector-specific sensorimotor information. These findings support a largely effector-independent MA mechanism, consistent with motor-equivalence principles, that is flexibly reweighted toward effector-specific transformations under uncertainty, thereby linking low-level sensorimotor corrections with abstract motor representations and consolidating a gap in conceptual frameworks of the sense of agency.

## Highlights

• Motor awareness for everyday actions relies on effector-independent representations
• MA thresholds and sensitivity indicate a shared, underlying representation
• Under sensorimotor conflict, MA shifts toward effector-specific representations
• MA recruits effector-independent representations that enable motor equivalence

## Graphical abstract

**Figure:**
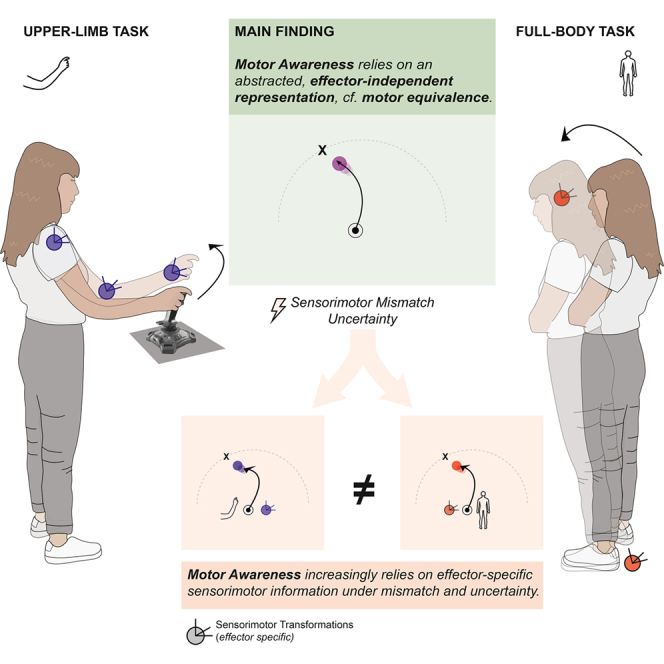

## Introduction

We perform most of our daily activities without paying particular attention to the details of how an action is performed. When we prepare a cup of tea, perhaps the only aspect we consciously monitor is when we pour the hot water into the cup. However, we are not generally aware of the particular grasp we choose to open the cupboard, nor of postural adjustments such as when we shift our weight onto one leg in order to reach for the cup. Nonetheless, we maintain a sense of agency (SoA) and control over all of the aforementioned actions (even if this sensation is “phenomenally thin”^1^^,^^2^). It has been argued that motor awareness (MA), in other words our ability to consciously monitor the details of our actions, provides one potential limit of our SoA^3^^,^^4^; our SoA should not be more sensitive to perturbations than our ability to perceive and automatically adjust for them. Furthermore, a central underlying mechanism, from finger-related movements to full-body actions, has been proposed, making MA effector independent.^4^^,^^5^^,^^6^ This is in-line with recent evidence for an abstract action system that suggests effector-independent representations for e.g., reaching or grasping movements,^7^ extending the general notion of effector-independent motor representation,^8^^,^^9^ to effector-specific sensorimotor transformations. Here, we explicitly tested the effector-independence hypothesis for MA, and by extension SoA, by asking participants to the same, goal-directed reaching task with (1) their right hand (effector task 1) and (2) by leaning with their trunk (effector task 2). We hypothesized that MA thresholds would co-vary between body-part and full-body actions, despite their vastly different skeletomotor organization and different cortical and subcortical control mechanisms.^10^^,^^11^^,^^12^ To evaluate the robustness of effector-independent MA, we carried out two further experiments. First, we controlled for general differences in reaching performance between the hand and the trunk by asking participants to perform a blind reaching paradigm with the respective effector. Second, we introduced a dual task paradigm, to evaluate MA under cognitive loading; changes induced in MA and motor control, should be reflected and comparable across effectors, if the effector-independent hypothesis is correct.

Action monitoring and MA have been studied since the early work by Nielsen.^13^ These studies have been primarily focused on reaching movements performed with the arms^3^^,^^13^^,^^14^^,^^15^ and hands,^16^^,^^17^ or fingers.^18^ MA is generally evaluated in response to sensorimotor mismatches between visual and proprioceptive feedback and the motor commands and studies consistently demonstrated that participants attributed feedback that was deviated in space by up to 6.5°–15° to their own movement^15^^,^^19^^,^^20^^,^^21^ and even larger deviations in neurological patients^22^ and individuals diagnosed with schizophrenia or with a genetic predisposition for psychosis.^16^^,^^23^^,^^24^^,^^25^^,^^26^ Similarly, experiments with temporal delays between the participant’s actual hand and the visual feedback illustrated that participants self-attributed movements with up to 150–200 ms of delay.^23^^,^^27^

Aside from brief reaching movements, MA and the SoA have further been investigated for continuous movements such as finger-tapping using auditory feedback^18^^,^^28^ or hand opening and closing using visual feedback.^29^^,^^30^ Moreover, footsteps generated during walking, evoke similar psychometric responses in MA as reported for auditory feedback for upper-limb movement.^6^ More recently the research of Kannape et al.^4^^,^^31^ extended these paradigms by studying walking agency using visual feedback. The thresholds in these studies are again comparable to those involving only upper-limb movements both with respect to temporal and spatial characteristics. Taken together, these studies suggest that MA is independent of the end -effector as well as supramodal,^32^ arguing for a common action monitoring mechanism.^6^^,^^33^

MA is intrinsically linked to one’s actual, ongoing movement and its accompanying reafferences^34^ rather than a mediated action consequence such as an audiovisual stimulus generated via a button press, as commonly used in SoA paradigms. MA has therefore closely been associated with computational models of sensorimotor control, most notably the comparator framework.^35^ The framework relies on a comparison of reafferent feedback and a predicted future state to improve sensorimotor control. It further proposes that small movement errors are automatically corrected via this comparison, as has for example been observed when participants update their movement toward a target object displaced during a saccade.^36^ This mechanism which optimizes the estimation of our movements to correct them also been likened to a Kalman Filter within an optimal control framework.^37^^,^^38^ Larger errors on the other hand may be perceived by participants and consciously corrected as seen when spatiotemporal deviations exceed the MA threshold.^3^^,^^13^^,^^31^ Further evidence for the comparator and more generally the predictive coding framework,^39^ comes from research on sensory attenuation,^40^ which, relevant to the current study, has similarly been shown to be comparable for action consequences generated by both the hand and the foot.^41^ Therefore, this framework not only provides a model for online movement adjustments, both automatic and conscious, and sensory attenuation, but also a model mechanism to discriminate between our own actions from the ones generated by the environment^42^ or other agents.^20^ As this framework applies to most types of movements it also, in principle, supports the idea of effector-independent MA. However, no prior research has investigated MA for comparable movements achieved with two different effectors within a single study and cohort to test this hypothesis.

While co-varying MA thresholds across effectors are one indication that MA may rely on abstracted, effector-independent motor representations, we set out to “stress-test” this hypothesis by running the same paradigm while taxing cognitive resources. Our study is therefore not only the first to compare motor performance (MP) and awareness for distinct effectors in the same cohort and across matching paradigm but also the first to combine this with cognitive loading. This is important as cognitive loading, induced by asking participants to perform a secondary motor or cognitive task in addition to a primary one, has systematic effects on motor control.^43^ Dual tasking is regularly applied in clinical populations^44^ and the elderly,^45^ where particularly strong influences have been demonstrated on balance and locomotion. Previous research has also indicated an increased separation between MA and MP during dual tasking.^4^^,^^5^^,^^46^ During goal-directed walking this was reflected in the finding that cognitive loading did not alter the locomotor trajectories but impaired motor control (walking velocity) in general, but MA only in trials with high perceptual uncertainty. Understanding how MA is altered during dual tasking is not only relevant for clinical application but also may shed further light on how it differs for various types of movements and effectors. For that reason, a visual color-word Stroop task (Stroop^47^ 1935) was introduced for the movement of the full body and the hand in this study.

Finally, potential effector-dependent differences in MA relating to the spatial mismatches investigated here could also be due to baseline differences in reaching performance. E.g., higher MA thresholds for one effector may reflect an increased difficulty to perform a motor task, rather than a difference in the MA mechanism. In order to control for differences in hand- and trunk-based reaching, we asked participants to perform blind-reaching tasks both with their upper limb and by leaning with their full body. This is further motivated by a recent study suggesting differences in error and variability between the two effectors,^48^ suggesting that trunk-controlled movements may be better than hand-controlled movements, even though the latter are more common with respect to visually guided feedback.

**Figure 1:**
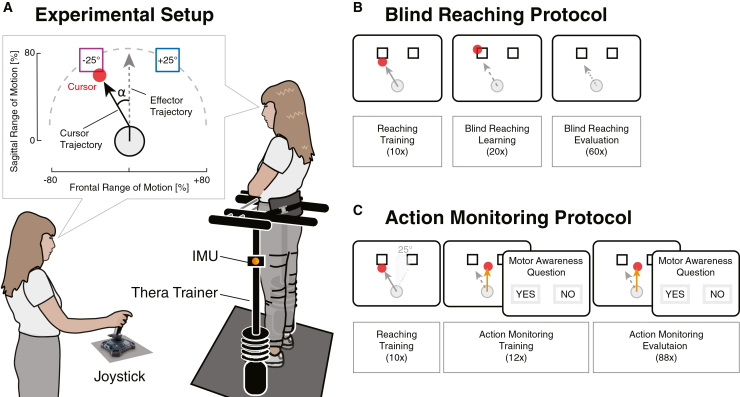
Setup (A) Experimental setup and visual feedback. Participants either controlled the cursor by moving the joystick with their hand or by leaning forward with their whole body, while supported by the Thera Trainer Coro, thus mimicking the movement of the joystick (counter balanced). In the action monitoring task, the cursor trajectory could be deviated by an angle (α) that participants had to compensate in order to reach the target. (B) Baseline, blind-reaching task. Participants started the experiment with 10 training trials with continuous, congruent visual feedback. They were then trained to reach the target with only terminal feedback. During the evaluation of reaching accuracy and variability, terminal feedback was provided every 6 trials. (C) Action monitoring task. Participants next performed the goal-directed reaching movement that could either be non-deviated (control trials) or deviated by up to 30°. After 10 non-deviated reaching and 12 practice trials, participants performed a block of 88 trials with randomized deviated and control trials. Based on this, MA thresholds were determined. Once both tasks had been completed with one effector, they would be repeated with the other effector.

The primary goal of this study was thus to test the hypothesis that MA is effector independent and therefore comparable between goal-directed movements of the hand (reaching) and the full body (leaning), cf. Figure 1A. We hypothesized that MA thresholds would co-vary between the two effectors suggesting that MA may be based on an abstracted motor representation as opposed to the effector-specific sensorimotor transformations. In addition to the visually guided movements of the MA task, we further controlled for baseline differences in sensorimotor control between the hand and trunk in a blind-reaching task. Finally, we investigated the effects of cognitive loading on MA and movement kinematics. If MA is modulated by cognitive loading, we hypothesized to observe comparable changes for both effectors in relation to changes in MP.

## Results

### Action monitoring

MA was defined as the ratio of yes responses out of all trials of the same deviation. In a first step, we evaluated if MA was affected by independent variables linked to the setup, namely the location of the targets or the direction of the deviation. While no significant effect was observed for target side (*p* = 0.07), there was a strong interaction between target side and deviation side (i.e., sign of deviation, *p* < 0.001). MA was less sensitive, that is, yes responses in deviated trials were significantly more frequent, when the deviation side converged toward the target side. In other words, fewer experimentally induced deviations were noticed, when motor compensation was toward the center or midline. Given this interaction, we did not collapse the trials across targets and absolute deviation (cf. Kannape et al.,^5^ Kannape et al.,^31^ and van Elk et al.^46^). Instead, as the effect was symmetrical around both targets, we grouped the trials by mode: diverging, for negative or conflicting deviations, and converging, for positive or “helpful” deviations; control trials with no deviation were analyzed separately.

**Figure 2:**
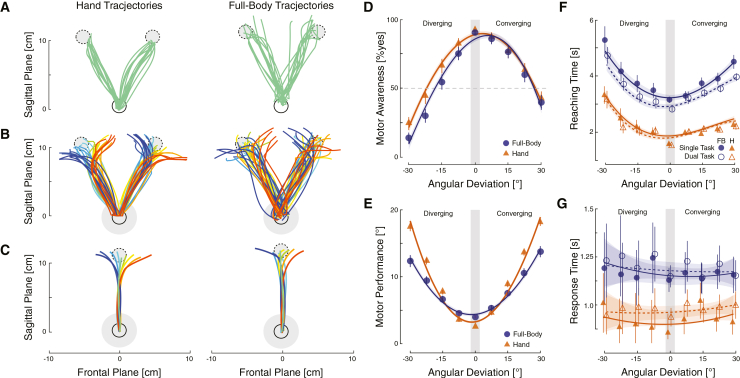
Motor performance (A–C) Reaching trajectories of a single representative participant during the visually guided MA task. (A) Trajectories from the resting position (solid circle) toward the two target locations (dashed circle) in control trials were more accurate for the hand (cf. G). (B) Trajectories toward the targets in trials with deviations to the left (red tone lines) and to the right (blue tone lines). The shaded blue circle indicates the distance at which the deviations start. (C) Average trajectories of this participant collapsed across the two target locations. (D) MA was modulated by deviation angle as well as the interaction of target- and deviation angle. Participants made more attribution errors when the deviation angle converged with the target angle. (E) Averaged MP across participants for non-deviated trials in single and dual tasks. (F) Reaching times were faster for the hand than the full body but showed a similar pattern. Trials took longer to complete with increasing mismatches and when target location and deviation diverged. (G) Response times were faster for the hand than the full body but showed less modulation through the angular deviation. Data are represented as mean ± SEM.

MA ratings in control trials with no deviation should be high, as participants receive veridical feedback of their movements.^33^ Based on previous studies, this correct MA should also be robust to cognitive loading.^5^^,^^46^ In-line with these hypotheses, participants correctly self-attributed 89.9 ± 1% and 91.3 ± 1% (mean ± SEM) of the non-deviated trials for the full body and hand, respectively (Figure 2D, shaded area). We neither observed a main effect of task or effector, nor an interaction (*p* > 0.282). There is strong evidence in favor of H_0_, i.e., that these distributions do not differ between the two effectors (BF_10_ = 0.277, *p* = 0.283). As before, we used Bayesian analysis to show evidence in favor of H_0_ (overall BF_10_ = 0.340, *p* > 0.283): MA in control trials does not differ across tasks.

All participants clearly distinguished between the introduced deviations, as demonstrated by the significant effect of deviation (*p* < 0.001, see Figure 2D). With respect to the mode of the feedback, MA was significantly lower, that is more sensitive to deviations, when these were diverging (*p* < 0.024). MA was also more sensitive for the full body compared to the hand (effector, *p* < 0.001) and during the dual task in general (*p* = 0.014). Finally, a significant interaction between mode and effector (*p* = 0.001) was detected. This interaction was driven by a significant difference in diverging trials between the two effectors; MA was more sensitive for full body movements.

### MA thresholds and sensitivity

We fitted a psychometric function to the MA responses to extract the 50% threshold and the slope of the psychometric curve. The threshold represents the point of subjective equality (PSE) for the detection of the introduced deviation, indicating the stimulus strength at which participants respond “yes” or “no” with the same likelihood. The slope indicates the sensitivity to the stimulus, here the deviation. If sensitivity drives differences in thresholds, i.e., higher sensitivity leads to lower thresholds, this argues for a perceptual mechanism, whereas no correlation would suggest a response bias. The values are calculated per participant, condition, and effector as summarised in Table 1. This allows us to compare if thresholds and slopes, independently, correlate across participants. If the underlying mechanism is shared across effectors, we should further observe a shared relationship between slopes and thresholds at the group and individual level.

**Table 1:** Motor Awareness Psychometric Thresholds and Slopes

Effector	Task	Threshold (μ ± SD)	CI (95%)	Slope (1/σ)	CI (95%)
Hand	single task	26.81 ± 12.33	[21.04, 32.58]	0.077 ± 0.035	[0.061, 0.093]
Hand	dual task	22.86 ± 8.07	[19.08, 26.64]	0.07 ± 0.025	[0.058, 0.082]
Full body	single task	22.01 ± 9.21	[17.7, 26.32]	0.081 ± 0.040	[0.062, 0.1]
Full body	dual task	20.57 ± 6.77	[17.4, 23.74]	0.078 ± 0.027	[0.065, 0.091]

**Figure 3:**
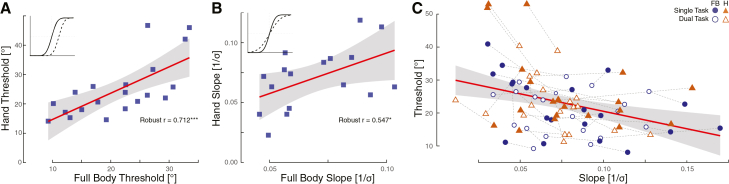
Motor awareness and psychophysics (A) Individual psychometric thresholds were calculated for each participant, effector, and task. Single and dual task thresholds were then averaged to have two thresholds per participant, hand and full body, respectively. These thresholds significantly correlated across participants. (B) The slope of the psychometric fits, indicating stimulus sensitivity, also correlates across participants. (C) There is a strong relationship between slopes and thresholds at the group and individual level. Participants with higher sensitivity generally displayed lower thresholds, suggesting that responses are driven by a common underlying mechanism rather than a bias in response patterns. Robust correlation: ∗*p* < 0.05, ∗∗*p* < 0.01, ∗∗∗*p* < 0.001.

We observed that MA thresholds significantly correlated between the hand and full-body conditions across participants (robust r = 0.712, *p* < 0.001), Figure 3A. Similarly, sensitivity, as expressed by the slope, showed a significant correlation between effectors (robust r = 0.547, *p* = 0.0125), Figure 3B. The linear mixed model, expressing MA thresholds as a function of sensitivity, revealed a significant main effect of sensitivity on thresholds (F(1, 13.55) = 9.89, *p* = 0.007), indicating that higher sensitivity (steeper slopes) was associated with lower thresholds, Figure 3C. There was also a significant main effect of effector (F(1, 54.78) = 12.72, *p* < 0.001), with thresholds differing between hand and full-body conditions. The main effect of task did not reach significance (F(1, 53.29) = 3.31, *p* = 0.074). The interaction between slope and task was not significant (F(1, 54.20) = 0.88, *p* = 0.354), indicating that the slope-threshold relationship was consistent across single and dual task conditions. There was a significant interaction between slope and effector (F(1, 55.38) = 6.32, *p* = 0.015), suggesting the strength of the relationship varied slightly between effectors. However, the three-way interaction among slope, task, and effector was not significant (F(1, 54.50) = 0.19, *p* = 0.668), indicating no evidence of complex moderation.

**Figure 4:**
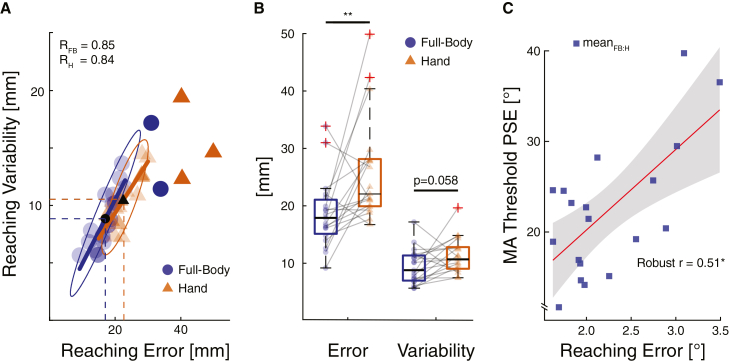
Blind reaching (A) Blind reaching error and variability of each participant and for the full body (blue dots) and the hand (orange triangles) calculated across the 60 blind reaching trials for each participant. Opaque samples represent outliers; the two ellipses are the robust data centers delineating the 95% bootstrapped confidence interval. The outliers are not excluded from further analysis. (B) On average (black markers), blind reaching with the full body leads to more accuracy (smallest reaching error) and more precision (lowest variability), compared to blind reaching with the hand. (C) Averaged errors in the blind reaching task correlated positively with averaged MA thresholds, indicating that participants with better blind reaching performance more readily judged deviated feedback trials as not corresponding to their actual movement. However, this was not significant at the level of hand or full-body movements individually. ∗*p* < 0.05, ∗∗*p* < 0.01, ∗∗∗*p* < 0.001. Data are represented as mean ± SEM.

Finally, we investigated whether there was an association between the participants’ ability to monitor their action, as expressed by the PSE, and their reaching accuracy derived in study 1 (cf. Figure 4C). This could account for potential differences observed in MA. We conducted a robust correlation analysis to examine the relationship between overall perceptual thresholds and average reaching error across participants, collapsed over task and effector. The analysis revealed a significant positive association (*r* = 0.51, *t*(17) = 2.44, *p* = 0.026), indicating that participants with greater reaching error tended to exhibit higher perceptual thresholds. However, this correlation was not evident when individually examining hand and full-body reaching errors with single task perceptual thresholds (both *p* > 0.200).

### MP

Figure 2A shows the trajectories of the hand and full body in the sagittal plane from the resting position to each of the two targets for non-deviated trials. Visually guided reaching performance for non-deviated trials, independent of the task, was similar across participants and significantly better for the hand, contrary to the blind reaching data (main effect of effector, *p* < 0.001, error_hand_ = 2.5° ± 0.06; error_full-body_ = 4.0° ± 0.1, mean ± SEM, see Figure 3E gray shaded area). We observed a significant reduction in reaching error during dual tasking independent of the effector (main effect of task, *p* = 0.01). There was no interaction between factors task × effector for non-deviated trials (*p* = 0.38).

All participants adapted their reaching movements to account for the target and angular deviations (Figure 2E). Thus, trajectories were deviated in the opposite direction of the angular deviation, as reported for goal-directed tasks for the hand^3^^,^^49^^,^^50^ and while walking.^5^^,^^31^ This compensation significantly increased with the experimentally induced angular deviation (main effect of deviation (*p* < 0.001). MP was also significantly higher when the compensation was toward the center, i.e., when the deviation side converged toward the target side (see Figure S1B). Therefore, a main effect of mode was detected (*p* < 0.001), as well as main effect of task (*p* < 0.001). As for control trials, dual tasking significantly improved MP. An interaction between deviation and effector was noticed (*p* < 0.001). Motor compensation was higher for the full body during the smallest deviation (which may reflect lower accuracy during visually guided movements), while motor compensation was better for the hand during larger deviations (>15°).

### Blind reaching

We evaluated blind-reaching performance as a baseline performance metric between effectors. The blind-reaching error, representing reaching accuracy, was on average 26 ± 9 mm (mean ± SD) for the hand and 18 ± 6 mm for the full body, while the effectors presented a variability, i.e., precision, of 11 ± 3 mm and 9 ± 3 mm, respectively (Figure 4A). These two metrics are highly correlated for the two effectors (Pearson’s skipped R > 0.84) as participants with small reaching errors (accuracy) also displayed low reaching variability (precision). The average blind-reaching error was significantly lower for the full body than for the hand (paired *t* test, *p* = 0.007). Although reaching variability was higher for the hand than for the full body, this difference did not reach significance (paired *t* test, *p* = 0.058) (see Figure 4B). The average reaching time across trials was shorter for the full body than for the hand (paired *t* test, *p* = 0.007, Figure 2F).

### Response times, reaching times, and dual task performance

We observed a significant difference in response times depending on the effector (*p* < 0.001) as participants answered significantly faster when the effector was the hand. Response times were not significantly affected by the magnitude of the deviation (*p* = 0.34).

Reaching times for control trials were significantly shorter for the hand than for the full body (*p* < 0.001). Dual tasking led to shorter reaching times for the full body, whereas they remained similar for hand movements (*p* = 0.001). Reaching time significantly increased with the introduced deviation (*p* < 0.001). As for control trials, reaching times were significantly higher for the full body than for the hand (*p* < 0.001). Dual tasking significantly decreased reaching duration (*p* < 0.001). The reaching time was lower when the deviations were converging (*p* < 0.001).

During the dual task, participants named the wrong color of the target on average 0.74 ± 0.2 times (mean ± SEM) over 88 trials. No significant difference was detected for the two effectors (paired *t* test, *p* = 0.71).

### Results summary

The key findings we report here are that all participants successfully completed the MA task by compensating for the introduced angular deviations (MP). MA in undeviated control trials was uniformly high and did not differ across effectors and tasks. Similarly, MA ratings systematically decreased as introduced deviations increased. There was an interaction between deviation angle and target position that facilitated movements in converging trials, where movement compensation toward the midline was needed and impeded them in diverging trials, where movement compensation toward the outside was required. In MA this was reflected by more attribution errors in converging trials. Unlike in control and converging trials, MA in diverging trials differed between hand and full-body movements and were more accurate for the former. Psychometric functions were fit to the data to extract MA thresholds and slopes, indicating the point of subjective equality and stimulus sensitivity, respectively. These correlate strongly across and within participants, and, as confirmed by mixed methods analysis, as participants with higher sensitivity generally demonstrated lower awareness thresholds.

## Discussion

A central challenge toward understanding our SoA from a sensorimotor account has been to quantify to what extent we are, or can be, aware of the details of our movements.^34^^,^^51^ Evidence from seminal case reports e.g., Goodale et al.^52^ as well as behavioral studies has pointed to a dissociation between our movements and our awareness thereof,^53^^,^^54^ even when prompted to consciously monitor these (cf. introduction). Given the similar MA thresholds observed across tasks, effectors, as well as feedback modalities, it has been argued that there is an underlying, supramodal, and effector-independent monitoring mechanism^33^ underlying our SoA. To test this hypothesis, we here assessed MA thresholds for goal-directed hand- and trunk movements within a single group of participants. Based on our findings, we propose that the central nervous system does make use of a general strategy to monitor goal-directed movements of our hand or the trunk and that this is in the majority of cases based on abstract movement representations, rather than a direct comparison of effector-specific sensorimotor predictions and their reafferents. In-line with this, our results support the effector-independent hypothesis as we observed corresponding changes in MA depending on kinematic task demands (Figure 2D), under cognitive loading, as well as a strong co-variance of deviation sensitivity and perceptual thresholds within participants (Figure 2D). That said, we also observed effector-specific differences, notably in diverging trials, indicative of a shift in strategy in case of kinematically more demanding tasks. We discuss our findings in relation to effector-independent representations in the motor domain and how such abstracted representations may play a role in understanding MA as the key sensorimotor component of our global SoA and the experience of the self as an intentional agent.

In the current study, we made several observations that support an effector-independent mechanism underlying our SoA. To begin with, MA ratings in control trials matched across both effectors and conditions, suggesting that there are no differences for MA at baseline and by extension the SoA, which roughly translates to everyday situations without significant external perturbations. The fact that we observed differences between effectors in MP for blind reaching and visually guided reaching gives an indication of the inherent tolerance of the mechanism and links to the aforementioned dissociation between action and perception; although full-body movements were more accurate and precise for blind reaching and hand movements were superior for visually guided reaching, SoA did not differ between the two effectors. These results also extend our findings for goal-directed walking^5^^,^^46^ and continuous walking^4^ where, as observed in the current tasks, MA ratings in control trials were not affected by cognitive loading, independent of its detrimental effects on MP.^43^^,^^44^

Further evidence for effector independence comes from deviated trials, where body-part and full-body MA thresholds and deviation sensitivity showed a strong correlation within participants: participants that were better at detecting experimental deviations of their hand movements also picked up on more of the same deviations during full-body movements. Furthermore, there is a clear relation between sensitivity and thresholds as participants with higher sensitivity systematically had lower thresholds. Our findings are consistent with a shared perceptual mechanism rather than a condition-specific or bias-driven explanation. Extending this similarity, we observed clear and corresponding effects of kinematic task demands on MA ratings for the hand and full body in converging trials, where participants had to compensate for the experimental deviation by moving toward a central location, straight ahead. When the experimental deviation “aided” the movement in this way, MA thresholds significantly increased for both effectors. That is, fewer mismatches were detected. While these two findings are in-line with an effector-independent mechanism, the fact that they persist even though we observe effector-specific differences in motor compensation may point to a more general commonality, namely that of an abstract motor representation serving as a basis for the monitoring mechanism. As we observed, particularly for walking under cognitive loading,^5^^,^^46^ conscious monitoring, unlike the continuous automatic adjustment observed for sensorimotor control, may rely on a movement representation formed in the motor planning phase before the onset of the movement.

As previously proposed,^33^^,^^55^ a goal-directed paradigm in itself may create a type of bias linked to the successful completion of the task as determined at the planning stage: MA ratings are consistently higher for successfully completed trials even if sensorimotor reafferents about the on-going movement contain a strong error signal.^32^ In addition to the “limits of conscious action monitoring” described in Fourneret and Jeannerod^56^ and already evident in Nielsen,^13^ the evidence for such a bias when comparing converging and diverging trials goes beyond simple visual dominance.^57^^,^^58^^,^^59^

What happens when the combination of the target location and the introduced deviation make it more difficult to reach the target? Although we report matching MA thresholds in non-deviated and converging trials, independent of underlying sensorimotor differences, MA ratings differed in the case of diverging trials (Figures 2D and S1A). In this case, the introduced deviation is toward the inside of the target, i.e., the midline, such that compensatory movements must be made to the outside, i.e., the periphery. As it became kinematically more challenging to complete the task, MA ratings became increasingly more sensitive to the introduced deviations. MA thresholds were significantly lower, indicating that participants were more likely to reject even slightly deviated trials. This effect was evident across both effectors but especially pronounced for full-body movements. Overall, this change in MA thresholds in diverging trials is not surprising. As the motor task becomes more difficult, reaching accuracy decreases, meaning that visual errors at the endpoint are larger, on top of the increasingly conflicting proprioceptive reafferents. As the target is not clearly reached, even with increased effort, there may be less incentive to consolidate this conflicting information.

The more pressing question is why differences in MA thresholds between full-body and hand movements appear in these trials. As before, the answer does not appear to primarily lie with participants’ MP. The difference observed in MP was also evident in converging trials. It appears then that in case of exacerbated sensorimotor conflict, the proposed effector-independent mechanisms may be augmented, or reweighted, with respect to effector-specific sensorimotor information similar to a reliance on prior knowledge under uncertainty.^60^ Two possible explanations come to mind. For one, in the case of full-body movements the vestibular system may provide an additional, more relied upon cue of heading information, in particular when the deviated visual feedback becomes less reliable.^61^ This is in-line with the improved reaching accuracy and lower variability we report for blind reaching with the full-body. Furthermore, the decreased variability for trunk-related movement reported in that study^48^ is expressed in terms of improved muscle-activation patterns. This could potentially reflect an advantage of full body, multi-effector compared to single-effector movements with respect to optimizing variability and effort across larger muscle groups,^62^ with the latter also being linked to a modulation of agency ratings.^63^ As this non-visual representation of the full-body movement appears to be more accurate and potentially require relatively less effort than its hand-equivalent, it may more strongly influence MA ratings in diverging trials leading to the differences between effectors. For another, and tying into this explanation, such a reweighting would also be indicative of a shift in monitoring from an abstract motor representation involved in motor planning, to a stronger reliance on effector-specific reafferent feedback during motor execution,^64^ which as recent evidence suggest may be implemented simultaneously.^65^ White and Diedrichsen’s series of experiments lends further support to this explanation, suggesting that flexible changes in feedback control gains are crucial for corrective movements across multiple effectors, as opposed to separate principles.^66^ Anecdotally, their participants’ reports of being unaware of feedback-manipulations and attributing their poor performance to a lack of ability closely mirror the reports of Nielsen’s participants,^13^ once again highlighting the limits of our conscious access to these details. Future studies could investigate such a reweighting systematically by changing the reliability of visual, vestibular, and proprioceptive information when assessing MA.^61^^,^^67^

The notion of abstract motor representations is not a new one. Theories of sensorimotor control have long considered effector-independent components across actions based on concepts such as “motor primitives” and “motor equivalence”.^8^^,^^9^^,^^68^ Therefore, paradigms that put an emphasis on the content of the movement itself provide a unique opportunity to study the hypothesis that MA and the SoA rely on similar abstract motor representations. In their paper on an effector-independent action system, Liu and colleagues^7^ discuss the example of letters written with different body parts^68^^,^^69^ as a further example of motor equivalence (see also the accompanying commentary by Goodale^70^). This is similarly interesting from a conceptual standpoint for MA as e.g., writing the letter “a” with your foot, although now more closely tracked than for a simple reaching movement or while writing the letter with your hand, will still be evaluated to the notion of the letter “a” rather than an explicit sensorimotor transformation. Indeed, this was observed in experiment 3 of Knoblich and Kircher,^14^ where figural discrepancies that changed the shape of one’s ongoing drawing significantly increased awareness of sensorimotor mismatches compared to velocity mismatches that maintained the shape but delayed the feedback. One could say *function follows form* in MA.

How do these behavioral findings map onto neural networks and neurological conditions? An effector-independent motor system can be framed as an abstract, goal-level layer that encodes intended outcomes (e.g., “move the cursor to the target”, “write my signature”) in a format that can be flexibly realized by different effectors; neuroanatomically this scheme centrally engages premotor circuitry, presupplementary and supplementary motor areas (pre-SMA/SMA), and dorsal premotor cortex (PMd), for high-level plan formation and prediction,^71^^,^^72^ while the cerebellum supplies precise forward estimates. Parietal regions, notably the inferior parietal lobule (IPL), temporoparietal junction (TPJ), and superior parietal lobule (SPL) transform reafferent sensory signals into goal coordinates and detect mismatches.^73^^,^^74^ Motor equivalence provides the low-level implementations that allow an abstract plan to be expressed through distinct effectors, thereby linking high-level intention to effector-specific execution.^9^^,^^69^

Disruption of these networks has been linked to two clinical syndromes that exemplify a double dissociation between MA and SoA. First, anosognosia, a failure to acknowledge or recognize contralesional paralysis despite clear motor loss, is classically associated with right-hemisphere IPL/TPJ and adjacent ventral networks and with premotor tract disconnection in hemiplegia patients.^73^^,^^75^ Second, anarchic (or “alien”) limb, manifested in the performance of purposeful, often conflictual limb actions experienced as not self-generated, is most often reported following medial-frontal/SMA lesions, anterior callosal damage, or adjacent premotor/parietal pathology.^76^ These syndromes are parsimonious with an effector-independent, hierarchical comparator account and highlight the role of the premotor network (whose disruption is often necessary but typically not sufficient for persistent anosognosia).^74^^,^^75^ Their frequent upper-limb presentation is readily explained by somatotopic anatomy, lesion distribution (and to some extent reporting biases) rather than by absence of effector-independent planning. Complementary neurostimulation work shows that transient disruption of premotor/pre-SMA regions alters MA and implicit indices of agency, supporting the premotor layer’s direct role in conscious monitoring.^71^^,^^72^ Finally, partial-arousal states such as sleepwalking demonstrate that well-learned whole-body locomotor routines can run with attenuated (pre-SMA) monitoring and thus produce complex actions outside full conscious awareness.^46^

Indeed, evaluating continuous movements that are not inherently goal-directed may provide further insights into this characteristic of MA and the sensorimotor components underlying our SoA. In these studies, the movement itself is monitored, rather than a pre-specified outcome. The cyclical nature of movements such as opening and closing the hand,^77^ drawing,^14^ walking,^4^^,^^6^ or breathing^78^ already demonstrate examples of automatic sensorimotor synchronization that can coincide with or oppose MA ratings. Teasing out such quantifiable differences, how they may be abstracted in terms of motor primitives, equivalence, physiological requirements, figural descriptions, or explicitly desired states will be required to develop a clearer picture of the level of conscious access to our motor system as well as the sensorimotor contributions to our SoA.

Although the link between “low-level” sensorimotor control and “high-level” cognitive intentions and their importance for our sense of agency is intuitive and has long been the subject of research, conceptual accounts^51^^,^^79^ and computational models, originally designed purely for sensorimotor control,^80^^,^^81^ have thus far failed to provide a coherent framework. Notably, this issue arises at the level of abstraction, such that explanations have not been extrapolated from “inaccessible” sensorimotor control^51^ or relied on descriptive “top-down” and “bottom-up” interactions between categorically different types of representation^79^ (see also the hierarchical model for error detection^82^). Our current findings provide a common denominator for these approaches by showing that MA may well depend on sensorimotor transformation, as in the case of conflicting feedback, but additionally makes use of effector-independent movement abstraction already observed in the motor system. This “ignorance of the effector systems”^83^ has also been proposed as a prerequisite to understanding authorship and ownership for our thoughts in terms of internal models or predictive coding.^39^^,^^84^^,^^85^

What do we have in mind when we identify an on-going movement as our own, when we feel to be authors of our own actions? While previous frameworks have alternated between categorically different, body-part-specific sensorimotor transformations on the one hand to abstract intentions on the other, we propose that MA and our SoA generally rely on effector-independent representations or motor-abstracts. If there is a mediated action, as for example when flipping a light switch, its outcome may override internal representations about the actual movement. Similarly, under situations of high uncertainty or difficulty, we may have to focus on effector-specific transformations. However, most of our movements do not fall into these categories, yet we maintain a reliable sense of control over them and perceive correspondence (or at least no conflict) with our movement plans. Understanding MA as an effector-independent process that draws on abstract motor representations provides a first step, a proof by induction, to linking primary sensorimotor transformations and motor actions to abstract representations of our intentions and thoughts.

### Limitations of the study

While our results provide strong evidence for a largely effector-independent MA mechanism, our study comes with some limitations. First, participants’ movements are mapped onto a visual cursor. This means the design cannot fully disentangle to what extent participants base their responses on the cursor and visual error itself or on the underlying movement. While this is a general limitation of studies using spatiotermporal mismatches to study MA and the SoA,^33^ their findings have demonstrated that MA thresholds significantly differ from mismatch-detection paradigms in the same sensorimotor modalities.^86^ This highlights the role of both sensorimotor signals as well as cognitive contributions to MA (see also intentional binding^87^).

Second, our behavioral results could be corroborated and extended via further behavioral, neurostimulation, or clinical studies. For example, (1) a behavioral interlimb-transfer experiment could be used to evaluate if a novel mapping on one effector transfers to another effector^88^ based on an effector-independent representation; (2) a neurostimulation paradigm such as a transient disruption of pre-SMA or dorsal premotor cortex could more directly evaluate the role of premotor networks in MA^89^; (3) a clinical study with one of the patient populations discussed previously could evaluate the (un)availability of effector-independent representations. Addressing these limitations will help clarify the anatomical and computational basis of effector-independent MA.

## Resource availability

### Lead contact

Requests for further information and resources should be directed to and will be fulfilled by the lead contacts (olaf.blanke@epfl.ch and oliver.kannape@hug.ch).

### Materials availability

This study did not generate new materials or unique reagents.

### Data and code availability

• Data are available via OSF: https://osf.io/s63vw/.
• Code can be requested from the lead authors.
• For other enquiries, please contact the lead authors.

## Acknowledgments

This research was supported by two generous donors advised by 10.13039/100031252Carigest SA (Fondazione Teofilo Rossi di Montelera e di Premuda and a second one wishing to remain anonymous) to Olaf Blanke and the 10.13039/100009152Bertarelli Foundation to Olaf Blanke. The authors would like to thank Dr Mohamed Bouri and THERA-Trainer for providing the Coro platform for the study.

## Author contributions

Conceptualization, J.F., O.B., and O.A.K.; methodology, J.F., S.B., N.F., O.B., and O.A.K.; software, J.F. and O.A.K.; formal analysis and data curation, J.F., S.B., N.F., and O.A.K.; investigation, J.F.; resources, O.B.; original draft, J.F., S.B., N.F., O.B., and O.A.K.; review and editing, J.F. and O.A.K.; visualization, J.F. and O.A.K.; supervision, O.B. and O.A.K.; funding, O.B.

## Declaration of interests

The authors declare no competing interests.

## STAR★Methods

### Key resources table

**Table:** 

REAGENT or RESOURCE	SOURCE	IDENTIFIER
**Software and algorithms**		

MATLAB	NA	https://ch.mathworks.com/products/matlab.html
R	NA	https://cran.r-project.org

**Other**		

Thera-trainer	Thera Trainer Coro	https://www.thera-trainer.de/en/thera-trainer-products/standing-balancing/thera-trainer-coro/

### Experimental model and study participant details

Twenty-two healthy adults were enrolled for this single-group study (15 females, mean age = 25 ± 3.9 years, height = 1.66 ± 0.07 m) using convenience sampling. The sample size was determined based on the large effect size of the spatiotemporal mismatches reported in prior studies.^4^^,^^6^^,^^23^^,^^90^ This sample is large enough to be indicative of a dual-task effect and motivate an appropriately confirmatory study if desired. Due to the wide range of secondary tasks available and mixed findings across different participant cohorts, it is difficult to determine a meaningful sample size *a priori*.

Participants had intact or corrected to normal vision, intact musculoskeletal system, and no history of orthopedic, neural or psychiatric conditions. The experimental procedure described in this study was approved by the local ethics committee in accordance with the ethical standards laid down in the 1964 Declaration of Helsinki.

Two participants were discarded from the data analysis. The first one could not complete the balance task since they felt dizzy. They were the only participant to report vertigo. The second exclusion was done post-analysis, since the motor awareness threshold of this participant was below the point of subjective equality for all deviations, including control trials with no deviation.

### Method details

For the full-body configuration, participants were secured via straps at the pelvis level inside the THERA-Trainer coro,^91^ a therapy device for dynamic balance exercises used to track the movement of the full-body. Setup takes approximately 3 min and participants are securely positioned in a standing position, preventing potential falls. Its frame is height-adjustable and composed with two spring elements, whose resistance was set to minimal, for this study. The behavior of the spring system is comparable to the one of a joystick, meaning that it always pulls back toward the center. No degrees of freedom were locked during the experiment. It is equipped with an inertial measurement unit (IMU) which wirelessly transmits the tilt of the frame at a sampling rate of 30 Hz. The projection of the center of mass (CoM) in the frontal and sagittal direction was estimated by multiplying the pelvis height with the sinus of the roll and pitch angles given by the IMU, respectively. The upper frame of the THERA-Trainer core was modified to host a Logitech ATTACK 3 joystick, whose position was also streamed at 30 Hz.

#### Protocol

This study was divided in two parts. It started with 1) a blind reaching experiment, where participants were asked to reach a target without any visual feedback, and 2) an action monitoring study allowing the assessment of participants’ MA threshold. The order of the studies were not counterbalanced to avoid a potential carry-over effect of the visuomotor conflicts present in the action monitoring task to the blind reaching task. Participants folded their arms across their chest when leaning with their full body.

##### Study 1: Blind reaching

This study was composed of two experimental blocks; a full-body and a hand blind reaching block effectuated in a randomized order. A cursor representing either the projection of participant’s CoM or the hand position in the transversal plane was displayed on LCD monitor. Each trial started from the center target representing the resting position (i.e., standing straight or center of the joystick). A virtual target appeared on the top of the screen at one of two randomized locations. The two target locations were placed at ±25° from medial axis along a circle with a radius of 80% participant’s range of motion. This was calculated by multiplying the sine of the maximum tilt angle of the platform (8.5°), by the participant’s pelvis height. Participants were asked to perform a hand movement with a joystick or to lean with their full-body to reach the target. During the full-body block, they were instructed to keep the feet shoulder-width apart, to maintain a static base of support. For the hand block, they used their dominant hand.

All trials began from the resting position. The experiment started with a training phase where they were asked to reach one of the two virtual targets with congruent visual feedback. In the second phase (blind reaching learning), the cursor disappeared once the virtual target was presented. Participants were asked to reach blindly the virtual target, stop and stay once they think they reached the virtual target. They received terminal visual feedback of the cursor position to show them their reaching accuracy. They had 20 trials to improve their reaching accuracy. In the last phase, the terminal visual feedback appeared every 6 trials only.

##### Study 2: Action monitoring

Participants performed two experimental blocks, a full-body (FB) and a hand (H) action monitoring block, in randomized order. These two blocks were divided in two sub-blocks, comprising a single task (ST) and a dual task (DT). The sub-blocks order was randomized within blocks. The experimental procedure is illustrated in Figure 1B.

Our action monitoring task was similar to the paradigm used in previous studies to test for motor awareness through perceptual visuomotor conflicts of voluntary movements.^56^ As in study 1, participants were able to control a cursor either with their hand or by tilting the body. In both cases, they were asked to produce a trajectory as straight and smooth as possible. After reaching the target, they returned to the resting position to start the next trials. Return movements to the resting position were not analyzed, since they are assisted by the spring system of the THERA-Trainer coro or of the joystick.

The experiment started with a training phase (20 trials) where participants familiarized themselves with the devices and the task. The cursor position was congruent with their movement. Participants then performed the action monitoring training (12 trials) and evaluation (88 trials). In some trials (75%) and beyond a distance of 250 mm from the resting position, the trajectory of the cursor was deviated either clockwise (+sign) or counterclockwise (-sign) by an angular rotation of 7.5°, 15°, 22.5° or 30°. The amplitude and the side of the deviation was randomized and evenly distributed across targets (88 trials per sub-block, including 24 control trials, i.e., no deviations, and 16 trials per deviation) as in.^31^ At the end of each trial, participants used the joystick for both blocks to answer a question displayed on the screen asking: “Did the movement you saw on the screen correspond to the movement of your hand/body?”.^23^

In the dual task sub-blocks, participants performed the same action monitoring task, while executing a visual color-word stroop task.^47^ A color name (e.g., BLUE printed in red) appeared inside the cursor along the trajectory at 8 different distances from the resting position, so that participants could not predict the onset. The font color always conflicted with the color name. Participants were asked to say the font color out loud as fast as possible and then reply to the MA question quoted above.

### Quantification and statistical analysis

Data were processed offline using MATLAB (MathWorks, Natick, Massachusetts, USA) and the R environment.^92^ For both tasks, we first analyzed the reaching trajectories. Those were smoothed with a 5-sample-window moving average filter, interpolated and finally averaged across targets and deviations for each participant, see Figures 2A–2C. Trials where participants restarted their movement (i.e., came back to the center and reached again) were excluded. No trials were excluded in the action monitoring task for the final participants.

#### Study 1: Blind reaching

The reaching error of each trial was defined by calculating the Euclidian distance between the trajectory endpoint and the target center. The error amplitude is a direct measure of the spatial accuracy. To quantify the overall accuracy of each effector, reaching errors were then averaged across participants. To evaluate the participants consistency in their performance, the reaching variability was calculated by taking the standard deviation of the reaching errors and averaged across participants to assess the precision of each effector, Figures 4A and 4B. A trial was considered complete when the participant moved less than 0.05 cm for 0.5 s and if the distance traveled from the starting point was greater than 5 cm.

#### Study 2: action monitoring

First, we analyzed the responses to the action monitoring question. Motor awareness (MA) was defined as the number of yes-responses out of all trials of the same deviation. Correct self-attribution or MA was a “yes” response for non-deviated trials and a “no” response for deviated ones, cf. Figure 2D. The MA threshold was determined by fitting a psychometric curve to the participants’ responses across the absolute deviations and extracting at the 50% point of subjective equality (PSE) and the slope at the PSE, see Figures 3A and 3B. The PSE therefore corresponds to the stimulus strength, here deviation, at which participants judge half of the trials as corresponding to their own movement and half of them of not. PSE was then averaged across participants.

Second, we measured the trajectory endpoint to assess motor performance (MP), Figure 2D. The trajectory endpoint corresponds to the effector position when reaching the target radial distance, which is not equivalent to the trial endpoint (i.e., center of the target). MP was defined as total angle compensated by the participants considering the endpoint of each movement trajectories and measured from the onset of the deviation, which was 25 mm from the resting position.

Finally, as additional variables, we analyzed the reaching and response time. Reaching time was calculated from movement onset to end of the trial, Figure 2F. Movement onset was defined by the radial velocity being greater than 2% of the maximum trajectory velocity. The response time corresponds to the time from the appearance of the question to the participant’s response via joystick button, Figure 4G. Although we recorded response times, participants were not asked to reply to the motor awareness question as quickly as possible. They also could move to target at a speed that was comfortable for them.

The aforementioned dependent variables were analyzed as functions of the independent variables such as Deviation, Deviation Side, Target Side, Task, Mode, and Effector. Deviation Side was defined as the direction of the deviations (i.e., clockwise or counterclockwise). The target side was the position of the target with respect to the midline (left or right). The mode defines if the deviation converged toward or diverged away from the target position.

#### Statistical analysis

Mixed-effects linear models were conducted on MA (logistic), MP, and the two observatory variables as well as the PSE (see Table S1). For MA, the first model evaluated the effect of Target Side on control trials (m1). Then, we tested the interaction Task x Effector also on control trials (m2). On deviated trials, first the interaction Target Side x Deviation Side was tested (m3). Then, deviations were split in two modes (converging and diverging). We tested the effect of Deviation (m4) and then their absolute values were taken. We tested the interaction Deviation x Mode as well as the effect of Effector and Task (m5). Finally, for our last model, we tested the interaction Mode x Effector (m6) since this was not possible in the previous model. Models m1 to m4 were applied to MP to evaluate the effects of independent variables. The interaction Deviation x Effector and the effect of Mode and Task were evaluated in another model (m5′) For all the above-mentioned models, participants were specified as a random (subject) factor, allowing for random slopes and intercepts.

Bayesian analysis was used to determine whether there was significant evidence to suggest that the yes-responses for control trials were from the same distribution for Effector and Task, as well for the MA PSE. Paired Student t-tests were used to assess significant differences between full-body/hand reaching accuracy and variability. Bonferroni’s correction was applied for multiple comparison. For correlations analysis, we used a robust procedure called Pearson’s skipped-correlations,^93^ Figures 3A–2C, 4A, and 4C. This method protects against bivariate outliers. We measured the strength of the association between average reaching error and PSE of each participant.
