# Supplementary material for: Motor equivalence in motor awareness
Source: iScience. 2026 Apr 21;29(6):115835. doi: 10.1016/j.isci.2026.115835 (PMC13194183; doi:10.1016/j.isci.2026.115835)
Supplement: Document S1. Figure S1 and Table S1 [file mmc1.pdf]

iScience, Volume *29*

Supplemental information

Motor equivalence in motor awareness

Jemina Fasola, Sophie Betka, Nathan Faivre, Olaf Blanke, and Oliver Alan Kannape

SUPPLEMENTAL INFORMATION

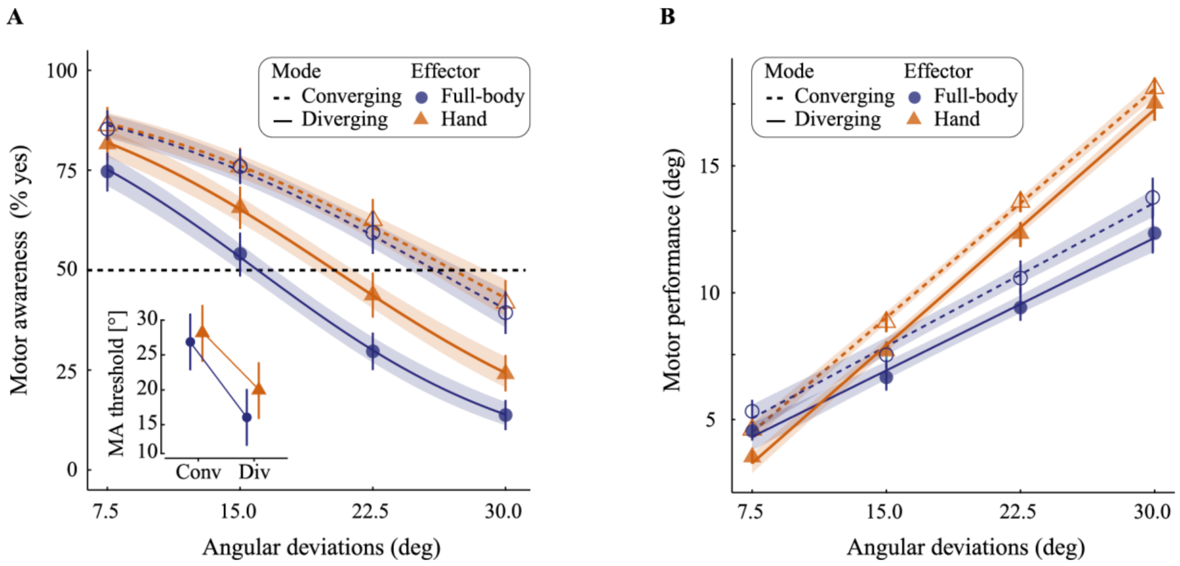

Supplemental Figure S1: Effect of kinematic demands on motor awareness (A) and motor performance (B). The two effectors have similar motor awareness thresholds for converging trials, while MA threshold was better for full-body during diverging trials. Motor performance is better for the hand and during converging trials.

Table S1 Statistical Analysis Detailed Results

Parameter mean±SEM	*Trials*	*Conditions*					*Model*	*Effects*	*p*
			**Left target**		**Right target**				
Motor	control		0.91±0.01		0.89±0.01		m1	Target Side	0.07
awareness		CW	0.52±0.01		0.65±0.01			Target Side	<0.001
(% yes-	deviated						m3	Deviation Side	<0.001
reponse)		CCW	0.66±0.01		0.44±0.01			Target Side x Deviation Side	<0.001
			**7.5°**	**15°**	**22.5°**	**30°**			
	deviated	CW	85.8±1.4	75.9±1.7	60.9±1.9	40.6±1.9	m4	Deviation	<0.001
		CCW	78.1±1.6	59.8±1.9	36.7±1.9	18.9±1.5			
			**ST**	**DT**	**Conv**	**Div**			
							m2	Task	0.43
	control	FB	90.6±0.3	89.2±0.3	NA	NA		Effector	0.65
		H	92.7±0.3	90.0±0.3	NA	NA		Task x Effector	0.54
		FB	53.9±0.5	54.1±0.5	65.0±0.5	43.0±0.5	m5	Absolute Deviation	<0.001
	deviated	H	63.2±0.5	57.2±0.5	66.6±0.5	53.7±0.5		Mode	0.02
								Task	0.01

								Effector	<0.001
								Absolute Deviation x Mode	<0.001
							m6	Mode	<0.001
								Effector	0.36
								Mode x Effector	0.001
			**Left target**		**Right target**				
Motor performance (deg)	control		3.3±0.09		3.1±0.08		m1	Target Side	0.09
								Target Side	<0.001
	deviated	CW	9.35±0.2		10.1±0.2		m3	Deviation Side	<0.001
		CCW	10.5±0.2		9.1±0.2			Target Side x Deviation Side	<0.001
			**7.5°**	**15°**	**22.5°**	**30°**			
	deviated	CW	4.9±0.1	8.2±0.2	12.1±0.2	15.9±0.2	m4	Deviation	<0.001
		CCW	4.03±0.1	7.2±0.1	10.9±0.2	14.9±0.3			
			**ST**	**DT**	**Conv**	**Div**			
								Task	0.01
	control	FB	4.2±3.2	3.8±3.3	NA	NA	m2	Effector	<0.001
		H	2.6±1.8	2.38±1.8	NA	NA		Task x Effector	0.38
								Abs Deviation	<0.001
								Mode	<0.001
	deviated	FB	8.5±6.6	9.1±6.6	9.3±6.6	8.2±6.5	m5'	Task	<0.001
		H	10.6±6.4	10.9±6.5	11.3±6.2	10.2±6.7		Effector	<0.001
								Abs Deviation x Effector	<0.001
			**ST**	**DT**	Conv	Div			
Reaching time (s)								Task	<0.001
	control	FB	3.1±1.2	2.8±0.8	NA	NA	m2	Effector	<0.001
		H	1.6±0.6	1.5±0.5				Task x Effector	<0.001
								Deviation	<0.001
								Task	<0.001
	deviated	FB	3.9±1.7	3.5±1.3	3.9±1.5	3.6±1.5	m5''	Effector	<0.001
		H	2.1±0.9	2.0±0.8	2.3±0.9	1.9±0.8		Mode	<0.001
								Deviation x Effector	<0.001
Response time (s)								Task	0.61
	control	FB	1.13±0.7	1.14±0.6	NA	NA	m2	Effector	<0.001
		H	0.85±0.7	0.94±0.5	NA	NA		Task x Effector	0.26
								Deviation	0.57
	deviated	FB	1.17±0.7	1.18±0.7	1.17±0.7	1.18±0.7	m5'''	Task	0.35
		H	0.93±0.6	0.98±0.5	0.95±0.6	0.96±0.5		Effector	<0.001
								Deviation x Task x Effector	>0.16
